# Perioperative opioid requirements of patients receiving sublingual buprenorphine-naloxone: a case series

**DOI:** 10.1186/s12871-019-0745-3

**Published:** 2019-05-08

**Authors:** Yvette N. Martin, Atousa Deljou, Toby N. Weingarten, Darrell R. Schroeder, Juraj Sprung

**Affiliations:** 1 Department of Anesthesiology and Perioperative Medicine, Mayo Clinic, 200 First St SW, Rochester, MN 55905 USA; 2 Division of Biomedical Statistics and Informatics, Mayo Clinic, Rochester, MN USA

**Keywords:** Anesthesia, Buprenorphine-naloxone, Pain, Sublingual administration, Suboxone, Surgery

## Abstract

**Background:**

Buprenorphine, a partial opioid agonist, displaces full opioid agonists from receptors and may impede surgical pain management. We report the effects of a sublingual formulation of buprenorphine-naloxone, Suboxone (*SL-BUP*), on perioperative pain management.

**Methods:**

We identified all adult surgical patients from December 31, 2004, to January 1, 2016, who received *SL-BUP* within 30 days prior to procedures performed with general, regional, or combined general/regional anesthesia. We recorded opioid use during the procedure, in the post-anesthesia care unit (PACU), and during the 24 h following PACU discharge. We also examined opioid use in those who continued *SL-BUP* until the day of surgery vs those who preoperatively discontinued *SL-BUP*.

**Results:**

Thirty-two patients were treated preoperatively with *SL-BUP*. Three patients had regional anesthesia only, and opioid requirements were case dependent. Requirements were minimal for creation of an arteriovenous fistula and high following knee replacement and cesarean section. Twelve patients received combined general/regional anesthesia, and 17 received general anesthesia only. Intraoperative and PACU opioid use in these 2 groups were not significantly different (*P* = .10 and *P* = .93, respectively). In both groups opioid use increased after discharge from the PACU, and remained comparable between the general and combined general/regional group through the first 24 h after PACU discharge (*P* = .78). Although median [interquartile range] 24-h opioid doses were higher among patients who discontinued *SL-BUP*, the difference was not statistically significant in the general anesthesia–only group (*SL-BUP* discontinued, 199 [110–411] mg IV-MEq [intravenous morphine equivalent] vs *SL-BUP* continued, 106 [58–160] mg IV-MEq; *P* = .15) or in the combined general/regional group (*SL-BUP* discontinued, 140 [100–157] mg IV-MEq vs *SL-BUP* continued, 100 [73–203] mg IV-MEq; *P* = .94).

**Conclusions:**

Regardless of the type of anesthesia used, physicians treating patients with *SL-BUP* must be prepared to administer large doses of opioids during the early postoperative period. No difference in opioid requirements was noted between patients who perioperatively stopped *SL-BUP* versus those who continued *SL-BUP*.

## Background

Buprenorphine is a partial μ-opioid receptor agonist with high receptor affinity and slow dissociation [1]. Because of the long half-life of buprenorphine, the peak-and-valley plasma effects seen with shorter-acting opioids are minimized [2]. Furthermore, buprenorphine exerts κ-opioid receptor antagonism, which minimizes the psychotomimetic and euphoric effects associated with opioid use [3]. These unique features are useful for treatment of chronic pain [4–6] and maintenance therapy for individuals with opioid use disorders [7, 8]. Buprenorphine is sometimes coadministered with naloxone, most commonly in the sublingual formulation (film or tablet) containing buprenorphine and naloxone in a 4:1 ratio (Suboxone [*SL-BUP*]; Indivior Inc). A combination of buprenorphine and naloxone reduces the potential for inappropriate use of buprenorphine because naloxone has poor bioavailability when taken sublingually [9]; therefore, only clinically insignificant amounts of naloxone are absorbed into the blood. However, when buprenorphine-naloxone is abused, by intravenous or intranasal administration, naloxone is an effective antagonist of buprenorphine [10].

Buprenorphine may have implications in perioperative pain management. Because buprenorphine has high receptor affinity [1], it displaces full opioid agonists from receptors. In addition, buprenorphine reportedly has an analgesic ceiling effect [11] and, therefore, less efficiently provides pain relief to patients after surgery with expected high pain acuity. The effect of *SL-BUP* on perioperative pain management has rarely been reported [12]. In the present study, we describe opioid use in patients preoperatively treated with *SL-BUP* and undergoing surgical procedures under general, regional, or combined general/regional anesthesia. Our aim was to describe opioid use in order to provide a layout for an individual case analysis. We hypothesized that opioid use is high among patients treated with *SL-BUP*, and that the use of regional anesthesia reduces opioid requirements.

## Methods

This study was approved by the Mayo Clinic Institutional Review Board in Rochester, Minnesota. Consistent with Minnesota Statute §144.335 subd. 3a(d), we included only patients who provided authorization for research use of their medical records. Patients gave their written informed consent to have their medical records reviewed for the research purposes.

To identify all adult surgical patients who received buprenorphine, we queried the hospital electronic medical record database using the proprietary *Data Discovery and Query Builder* tool to identify all patients who received any form of buprenorphine (Suboxone, buprenorphine, buprenorphine with naloxone) within 30 days prior to a surgical procedure requiring anesthesia resources between December 31, 2004, and January 1, 2016. Each identified medical record was manually reviewed by one of the authors (Y.N.M.) to confirm inclusion criteria.

### Data abstraction

Medical, surgical, and anesthesia records were electronically abstracted with previously described proprietary software [13]. Characteristics included type of surgery and anesthesia, *SL-BUP* prescription strength (daily dose), whether the patient discontinued *SL-BUP* therapy before surgery (we noted the number of days without therapy and included patients who stopped between 1 and 30 days preoperatively), and whether therapy was uninterrupted (ie, the patient received *SL-BUP* on the day of surgery). This information can be reliably retrieved from medical records. At Mayo Clinic, hospital admission protocols include medication reconciliation (ie, registered nurse records all preoperative medications including the time of the last preoperative dose taken). We recorded intraoperative opioid use, as well as opioid use in the post-anesthesia care unit (PACU) and through the 24 h after discharge from the PACU. All full agonist opioids administered preoperatively were converted to intravenous morphine equivalent (IV-MEq) [14]. The presurgical buprenorphine dose administered with the *SL-BUP* prescription was not recorded in total IV-MEq. We also recorded the highest verbal numerical pain score (VNPS) (range, 0–10) reported in the 24-h postoperative period and whether a pain consultation was requested. Typically in our institution, a pain consult is requested by surgical service when their standard management is ineffective in treating postoperative pain.

### Statistical analysis

Unless noted otherwise, findings from the statistical analyses are summarized as counts and percentages. Opioid requirements are summarized as median (interquartile range [IQR]) milligrams of IV-MEq. We analyzed opioid requirements by using log transformation and compared groups with the 2-sample *t* test. All tests were 2-sided, and *P* < .05 was considered significant. Analyses were performed with SAS statistical software version 3.4 (SAS Institute Inc).

## Results

### Study population

We identified 32 patients who underwent surgical procedures while being treated with *SL-BUP*. Of these, 19 received *SL-BUP* on the day of surgery, and 13 discontinued treatment 1 to 30 days before surgery (12 stopped the *SL-BUP* 1–7 days before surgery, and 1 patient 30 days before). None of the patients in present series received *SL-BUP* in PACU or within 24 h after PACU discharge. Tables 1 and 2 present anesthetic and surgical characteristics, patterns of *SL-BUP* use (including whether patients had it on the day of surgery, or exact number of days since the last *SL-BUP* dose was taken), and perioperative opioid requirements (intraoperative, in the PACU, from PACU discharge through 24 h after discharge, and total use during the first postoperative day). Patients received various operations and anesthetic management modalities (regional anesthesia only, combined general/regional anesthesia, and general anesthesia only).

**Table 1:** Anesthetic and surgical characteristics and opioid requirements of patients taking buprenorphine-naloxone (*SL-BUP*) who underwent surgical procedures with regional anesthesia only or combined general/regional anesthesia

Pt	*SL-BUP* Administration		Procedure (Anesthetic Agents) Year of Surgery	Regional Anesthesia Type and Drugs	Surgery Time, min	Opioid Administration, IV-MEq				Highest VNPS^¶^
	Daily *SL-BUP* Dose, mg^†^	Days without SL-BUP^a^				Intra- operative	PACU Only	24 h after PACU Discharge	Total IV-MEq^§^	
Regional Anesthesia Only										
1	8 (2)	0	Cesarean section, 2012	Spinal^b^ (Bupiv+Fentanyl)	57	12	74	133	219*	10^c^
2	8 (2)	0	Minor vascular, 2010	Axillary^b^ (Mepiv)	127	5	0	0	5	NR
3	16 (4)^e^	2	Major orthopedic, 2016	Spinal^b^ (Bupiv)	94	15	0	74	89	9^c^
Combined Regional + General Anesthesia										
1	2 (0.5)^e^	0	Thoracic (Iso), 2009	Epidural^d^ (Bupiv+Hydromor)	110	39	30	134	203^*^	10
2	4 (1)	0	Major orthopedic (Iso + N_2_O), 2014	Interscalene^f^ (Bupiv+Clon)	93	30	18	53	100^‡^	9
3	16 (4)^e^	0	Major orthopedic (Iso + N_2_O), 2014	Interscalene (Bupiv+Clon)	70	15	20	38	73	8
4	16 (4)	0	Minor orthopedic (Pro), 2013	Axillary^b^ (Bupiv)	88	5	0	0	5	NR
5	16 (4)	0	Major orthopedic (Sevo+N_2_O), 2015	Femoral^f^ (Bupiv)	168	40	10	94	143^*^	10^c^
6	24 (6)	0	Major orthopedic (Iso + N_2_O), 2014	Interscalene^f^ (Bupiv)	60	5	13	420	437	9
7	32 (8)	0	Major orthopedic (Iso + N_2_O), 2011	Epidural^d^ (Bupiv)	128	13	5	60	78^*^	8^c^
8	24 (6)	2	Major orthopedic (Des), 2011	Psoas^e^ (Bupiv)	74	10	40	0	50	4^c^
9	24 (6)	2	Major orthopedic (Des), 2012	Sciatic^d^ + femoral^d^ (Bupiv)	124	53	15	33	100	8
10	4 (1)	4	Major orthopedic (Iso + N_2_O), 2012	Interscalene^f^ (Bupiv)	120	40	8	110	157	10^c^
11	16 (4)^e^	7	Abdominal (Iso), 2014	Spinal^f^ (Hydromor)	115	20	15	105	140^*^	10^c^
12	24 (6)^e^	30	Major orthopedic (Iso), 2013	Interscalene^f^ (Bupiv)	93	25	0	241	266	10

Abbreviations: *IV-MEq* intravenous morphine equivalent; *NR* not reported; *PACU* post-anesthesia care unit; *SL-BUP*, sublingual formulation of buprenorphine and naloxone (Suboxone); *VNPS* verbal numerical pain score. Anesthetic agents and local anesthetics: Bupiv, bupivacaine; Clon, clonidine; Des, desflurane; Hydromor, hydromorphone; Iso, isoflurane; Mepiv, mepivacaine; N_2_O, nitrous oxide; Pro, propofol; Pt, patient; Sevo, sevoflurane

^†^Daily dose is shown as buprenorphine (naloxone) in milligrams; ^§^Total IV-MEq includes intraoperative, PACU, and 24 h postoperative IV-MEq, ^¶^Highest VNPS applies to 24 h after PACU discharge. *Ketorolac, multiple doses; ^‡^Ketamine, multiple doses;

^a^*0* indicates that *SL-BUP* was preoperatively administered without interruption (ie, administered on the day of surgery or the night before); ^b^Primary anesthetic, ^c^Pain consultation requested, ^d^Patient was preoperatively prescribed oxycodone, as needed (nonscheduled regimen); ^e^Catheter inserted for continuous administration of local anesthesia, ^f^“Single-shot” local anesthetic or opioid injection

**Table 2:** Procedural characteristics, and opioid requirements of patients who received long-term buprenorphine-naloxone (*SL-BUP*) therapy and surgical procedures with general anesthesia

Pt	*SL-BUP* Administration		Procedure Type (Anesthetic Agents) Year of Surgery	Surgical Time, min	Opioid Administration, IV-MEq				Highest VNPS^¶^
	Daily SL-BUP Dose, mg^†^	Days Without *SL-BUP*^a^			Intra- operative	PACU only	24 h after PACU Discharge	Total IV-MEq ^§^	
1	1 (0.25)	0	Major orthopedic, (Des), 2011	322	78	27	318	423^*^	9^b^
2	2 (0.5)	0	Major oral (Des), 2012	206	61	26	115	202^*^	9
3	8 (2)	0	Neurosurgery (Iso + N_2_O), 2013	161	36	20	105	160	7^b^
4	12 (3)	0	Neurosurgery (Iso), 2013	240	40	10	58	108	10
5	12 (3)	0	Minor dental (Des), 2013	47	18	32	55	105*	8^b^
6	16 (4)	0	Neurosurgery (Des), 2013	130	28	15	38	80	10
7	16 (4)	0	Abdominal ^c^ (Iso), 2014	191	0	0	58	58^*/‡^	8
8	16 (4)	0	Transplant (kidney) (Iso), 2010	251	69	5	55	129^*^	8
9	32 (8)	0	Neurosurgery (Iso + N_2_O), 2013	80	25	10	5	40	10
10	32 (8)	0	Neurosurgery (Des), 2013	122	15	0	0	15	5
11	8 (2)	1	Abdominal (Des), 2015	90	33	12	10	55	7
12	12 (3)	3	Major cardiac (Iso), 2013	338	179	ND	233	411^c^	10^b^
13	24 (6)	3	Gynecologic (Iso), 2009	332	60	18	33	110	10
14	8 (2)	4	Major cardiac (Iso), 2009	205	144	ND	111	254	10^b^
15	24 (6)^d^	5	Neurosurgery (Des), 2011	395	49	0	150	199	7^b^
16	24 (6)	6	Major cardiac (Iso), 2015	163	241	ND	282	522^c^	9^b^
17	6 (1.5)^d^	7	Neurosurgery (Iso + N_2_O), 2009	39	25	30	58	113	9^b^

Abbreviations: *ICU* intensive care unit; *IV-MEq* intravenous morphine equivalent; *ND* no data available because the patient was admitted to the intensive care unit after surgery; *PACU*, post-anesthesia care unit; *Pt* patient; *SL-BUP* sublingual formulation of buprenorphine and naloxone (Suboxone); *VNPS* verbal numerical pain score. Anesthetic agents: Des, desflurane; Iso, isoflurane; N_2_O, nitrous oxide

^†^Daily dose is shown as buprenorphine (naloxone) in milligrams; ^§^Total IV-MEq includes intraoperative, PACU, and 24 h postoperative IV-MEq; ^¶^Highest VNPS applies to 24 h after PACU discharge; *Ketorolac; multiple dose; ^‡^Ketamine, multiple dose

^a^*0* indicates that *SL-BUP* was preoperatively administered without interruption (ie, administered on the day of surgery or the night before); ^b^Pain consultation requested; ^c^Patient preoperatively received 1000 mg acetaminophen 4 times daily, 800 mg ibuprofen 4 times daily, and lidocaine patch (5%) daily. This patient intraoperatively received 30 mg ketamine and 30 mg ketorolac (no opioids were administered); ^d^Patient was preoperatively prescribed oxycodone, as needed (based on nonscheduled regimen)

### Perioperative opioid requirements of patients who received regional anesthesia only

Three patients had surgical procedures with regional anesthesia as the primary and only type of anesthesia. Two of these patients received a spinal block and 1 patient received an axillary block (2 were receiving and 1 was not receiving *SL-BUP*) (Table 1). One patient who underwent arteriovenous fistula repair with an axillary block received 5 mg IV-MEq for placement of the block but did not receive additional opioids postoperatively. In contrast, 1 patient who underwent cesarean delivery and 1 patient who underwent knee arthroplasty had large opioid requirements following discharge from the PACU (Table 1).

### Perioperative opioid requirements of patients who received general anesthesia only or combined general/regional anesthesia

**Fig. 1:**
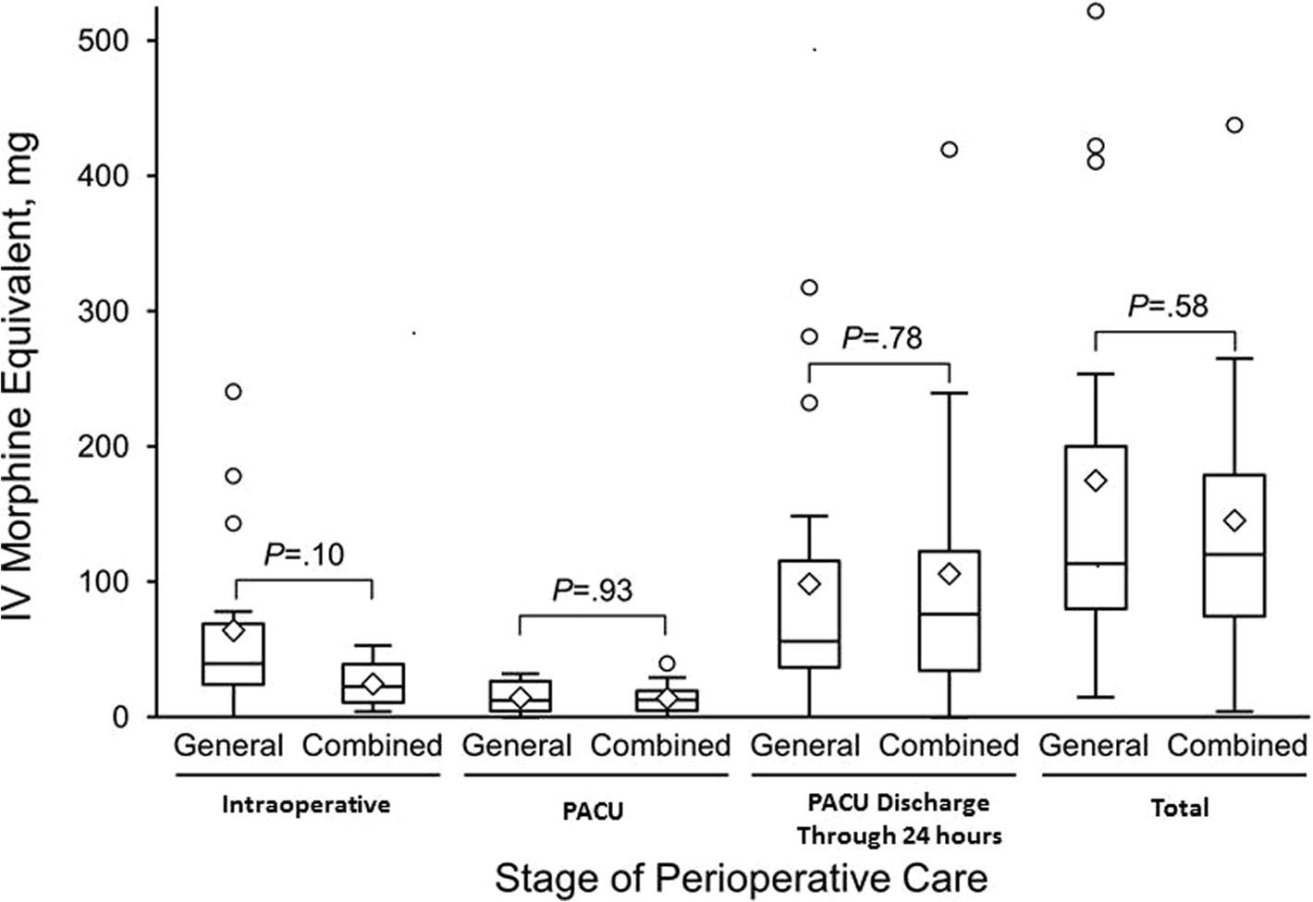
Intravenous morphine equivalents administered during the first postoperative day to patients who received *SL-BUP* according to stage of perioperative care. Plots separately show data for patients who underwent surgical procedures with general anesthesia only vs combined general/regional anesthesia. Intraoperative and postoperative IV-MEq (24 h after discharge from recovery room) were comparable between two anesthetic techniques. Box indicates interquartile range; middle line, median; diamond, mean; error bars, 1.5× interquartile range; open circles, outliers. IV indicates intravenous; IV-MEq, intravenous morphine equivalent; PACU, post-anesthesia care unit; *SL-BUP*, sublingual formulation of buprenorphine and naloxone (Suboxone)

Twelve patients underwent surgical procedures with combined general/regional anesthesia (7 were receiving and 5 were not receiving *SL-BUP*) (Table 1), and 17 patients underwent surgical procedures with general anesthesia only (10 were receiving and 7 were not receiving *SL-BUP*) (Table 2). Among these patients, perioperative opioid use was similar between patients who received general anesthesia only and those who received combined general/regional anesthesia (*P* = .58; Fig. 1). Intraoperative opioid requirements in the combined general/regional anesthesia group tended to be less than in the general anesthesia–only group; however, this difference did not reach statistical significance (*P* = .10). In the PACU, opioid requirements were similar between the 2 anesthesia groups (*P* = .93). In both groups, opioid use increased after discharge from the PACU, and total opioid administration was comparable between the 2 groups through 24 h after discharge from the PACU (*P* = .78). Table 3 shows opioid administration at different time points over the 24 h following surgery according to whether patients preoperatively continued or discontinued *SL-BUP*. Although median [IQR] 24-h opioid administration was higher among patients who discontinued *SL-BUP*, this difference did not reach statistical significance in the general anesthesia–only group (*SL-BUP* discontinued, 199 [110–411] mg IV-MEq vs *SL-BUP* continued, 106 [58–160] mg IV-MEq; *P* = .15) or in the combined general/regional anesthesia group (*SL-BUP* discontinued, 140 [100–157] mg IV-MEq vs *SL-BUP* continued, 100 [73–203] mg IV-MEq; *P* = .94). Postoperative pain consultations were requested for 8 of the 17 patients (47%) who received general anesthesia only, and for 6 of the 15 patients (40%) who received combined general/regional anesthesia (Fisher exact test, *P* = .74).

**Table 3:** Opioids administered for operations and during first 24hours postoperation to patients stratified by type of anesthesia and buprenorphine-naloxone (*SL-BUP*) use before surgery^a,b,c^

Time of Opioid Administration	General Anesthesia Only		*P* Value^d^	Combined General Anesthesia With Regional Block		*P* Value^d^
	*SL-BUP* Discontinued (*n* = 7)	*SL-BUP* Continued (*n* = 10)		*SL-BUP* Discontinued (*n* = 5)	*SL-BUP* Continued (*n* = 7)	
Intraoperative	60 [33–179]	32 [18–61]	.06	25 [20–40]	15 [5–39]	.33
PACU only	15 [6–24]	13 [5–26]	.98	15 [8–15]	13 [5–20]	.96
24 h after PACU discharge	111 [32–233]	56 [38–105]	.33	105 [33–110]	60 [38–134]	.86
Total 24-h	199 [110–411]^e^	106 [58–160]^f^	.15	140 [100–157]^e^	100 [73–203]^f^	.94

Abbreviations: *PACU* post-anesthesia care unit; *SL-BUP* sublingual formulation of buprenorphine and naloxone (Suboxone)

^a^Data are shown as milligrams of intravenous morphine equivalents; ^b^ Values are shown as median [interquartile range]; ^c^ Of 32 patients, 3 do not have recovery room data because of direct admission to the intensive care unit; ^d^2-sample *t* test after log transformation; ^e^Comparison of these data yielded *P* = .37; ^f^Comparison of these data yielded *P* = .90

## Discussion

Our main finding was that patients who received long-term treatment with *SL-BUP* had high opioid requirements through the first postoperative day, regardless of type of anesthesia used or whether *SL-BUP* was preoperatively continued or discontinued. Use of opioids increased substantially after discharge from the PACU, and by 24 h after PACU discharge, no differences in opioid requirements were noted between patients who underwent surgical procedures with general anesthesia only versus combined general/regional anesthesia. Because our case series describes a heterogeneous surgical population with variable use of *SL-BUP*, we cannot provide suggestions regarding the best opioid management practices for patients on *SL-BUP* undergoing surgery.

Importantly, the naloxone component of *SL-BUP* is clinically insignificant when taken sublingually; equianalgesic opioid doses formulated as *SL-BUP* versus other forms of buprenorphine only (eg, transdermal) are clinically similar. Therefore, this study evaluated the opioid requirements of individual patients and specific anesthesia management modalities related to buprenorphine use. Because buprenorphine tightly binds to opioid receptors and has an analgesic ceiling effect, management of more intense postoperative pain with buprenorphine may be difficult [11]. This difficulty may be further complicated by the long half-life of buprenorphine because the time needed for complete buprenorphine elimination may exceed the hospital stay. Thus, an individualized perioperative analgesic plan should be instituted for a patient receiving buprenorphine. Three regimens have been proposed: withhold buprenorphine for a period before surgery, continue buprenorphine use through the perioperative period, or increase the preoperative buprenorphine dose to a maximum of 32 mg/d [15].

To date, no unified guidelines exist regarding perioperative care of patients receiving buprenorphine. The rationale for withholding buprenorphine before surgery is based on the premise that buprenorphine levels on opioid receptors decrease over several days to the point at which high-affinity, pure μ-opioid receptor agonists (eg, fentanyl, alfentanil, morphine) exert clinical effects [11]. However, the optimal withholding time is difficult to determine primarily because buprenorphine elimination half-life varies from 16.4 to 42 h [11]. Given the expected drug-elimination rule (typically 5 half-lives), it would take 7.7 days for buprenorphine to be entirely eliminated from the body [11]. The additional difficulty of individualizing recommendations results from interpatient variability in buprenorphine elimination. However, even partial elimination, which could be achieved by withholding buprenorphine for 72 to 120 h, could allow for approximately 2 to 3 half-lives to pass and, in turn, improve the effects of full opioid agonists [15]. It has been suggested that patients should be offered full opioid agonists (eg, fentanyl patch or other traditional opioids) during the preoperative withholding period, if needed [15], which was the case for 7 of our patients. We have shown that patients who undergo surgery while receiving *SL-BUP* have high 24-h postoperative opioid requirements regardless of whether they preoperatively continued or discontinued *SL-BUP*. In the present study, we do not have a comparison group of opioid-naïve patients. However, we recently reported, in a case matched study, that patients not receiving long-term preoperative opioids received less opioid IV-MEq following discharge from the PACU compared to patients who underwent surgery while being on uninterrupted transdermal buprenorphine therapy (median [IQR] opioid dose of 15 [3–35] vs 54 [38–90] mg IV) [16]. Although patients included in the present study underwent different surgeries than those in our previous publication, and represent a mix of *SL-BUP* use (uninterrupted vs interrupted with varying time since last dose), the amount of opioids administered for these patients likely exceeds that expected for patients undergoing similar procedures and not receiving long-term opioids. In our case series, the use of the “single-shot” regional technique was not associated with reduced 24-h opioid requirements. Intuitively, regional techniques that use continuous infusion (eg, via indwelling catheters) may be more beneficial, but further studies are needed to confirm or refute this notion. Multimodal pain management (ie, nonsteroidal anti-inflammatory drugs, gabapentinoids, acetaminophen, clonidine, ketamine, and dexmedetomidine) may benefit patients receiving *SL-BUP*. However, postoperatively, multimodal pain management was rarely used in the present case series: ketorolac in 1 regional anesthetic, 4 combined anesthetics, and 4 general anesthetics; ketamine in 4 general anesthetics; and both ketamine and ketorolac in 1 patients in the combined anesthesia group. Doses of ketorolac ranged from 15 to 30 mg in the first 24 h, and ketamine doses ranged from 20 to 60 mg. Therefore, the role of multimodal pain management for patients receiving *SL-BUP* cannot be evaluated in the present study.

The rate of anesthesiology pain service interventions in the present series was 47% for patients who received general anesthesia and 40% for those who received combined general/regional anesthesia. These high rates are similar to that seen in our recent case-control study, where we demonstrated that patients on uninterrupted transdermal buprenorphine had a rate of 32% requiring pain consults, compared to 0% in the control group (no buprenorphine) [16].

The limitations of the study are related to its retrospective design. First, the small number of patients who underwent various surgical procedures, received different types of anesthesia, and received different doses of *SL-BUP* (which were either continued up to the day of surgery or discontinued 1–30 days before surgery) is a limitation that precludes generalization of our findings and observations toward the best practices for patients receiving *SL-BUP*. Second, intraoperative opioid use may be affected by knowledge that the patient is receiving *SL-BUP*, and therefore providers may preemptively increase opioid load to counteract postoperative pain. However, postoperative opioid needs are clearly guided by patient reports of pain, and therefore a high opioid load administered during the postoperative period truly reflects the increased opioid requirement of a patient receiving long-term *SL-BUP*. The number of patients in the regional anesthesia group is too small to make conclusions regarding the role of these techniques in reducing opioid requirements, especially since regional anesthesia management was not protocolized. In our practice, preemptive pain service management applies to patients with continuous regional anesthesia catheters (the anesthesia pain management team manages infusion rates and adjuvant analgesic therapies); and for those with single-shot regional block and those receiving general anesthesia only, the surgical service is primarily responsible for prescribing postoperative pain medications, and the anesthesia pain service is consulted on an as-needed basis only (ie, in cases with inability to control pain by primary service). However, retrospectively, we could not clarify whether pain consults were scheduled proactively under the assumption that patients were on *SL-BUP*, and therefore would require additional postoperative pain management, or if the consult was triggered postoperatively due to a true need for elevated pain management expertise.

## Conclusions

The opioid requirements of patients treated with *SL-BUP* were high and did not differ for those who underwent surgical procedures with combined general/regional anesthesia or general anesthesia only. No differences in opioid use were noted among patients who preoperatively continued or discontinued *SL-BUP*. However, our cohort was small and heterogeneous; hence, recommendations for perioperative opioid management for patients on *SL-BUP* cannot be made based solely on the present study.

## Abbreviations

IQR: Interquartile range
IV-MEq: Intravenous morphine equivalent
PACU: Post-anesthesia care unit
*SL-BUP*: Sublingual formulation of buprenorphine and naloxone (Suboxone)
VNPS: Verbal numerical pain score
